# Systematic analysis of *CNGA3* splice variants identifies different mechanisms of aberrant splicing

**DOI:** 10.1038/s41598-023-29452-9

**Published:** 2023-02-18

**Authors:** Peggy Reuter, Magdalena Walter, Susanne Kohl, Nicole Weisschuh

**Affiliations:** Centre for Ophthalmology, Institute for Ophthalmic Research, University of Tübingen, 72076 Tübingen, Germany

**Keywords:** Molecular biology, Genetics, Clinical genetics, Mutation

## Abstract

Achromatopsia is an autosomal recessive cone photoreceptor disease that is frequently caused by pathogenic variants in the *CNGA3* gene. Here, we present a systematic functional analysis of 20 *CNGA3* splice site variants detected in our large cohort of achromatopsia patients and/or listed in common variant databases. All variants were analyzed by functional splice assays based on the pSPL3 exon trapping vector. We demonstrated that ten variants, both at canonical and non-canonical splice sites, induced aberrant splicing, including intronic nucleotide retention, exonic nucleotide deletion and exon skipping, resulting in 21 different aberrant transcripts. Of these, eleven were predicted to introduce a premature termination codon. The pathogenicity of all variants was assessed based on established guidelines for variant classification. Incorporation of the results of our functional analyses enabled re-classification of 75% of variants previously classified as variants of uncertain significance into either likely benign or likely pathogenic. Our study is the first in which a systematic characterization of putative *CNGA3* splice variants has been performed. We demonstrated the utility of pSPL3 based minigene assays in the effective assessment of putative splice variants. Our findings improve the diagnosis of achromatopsia patients, who may thus benefit from future gene-based therapeutic strategies.

## Introduction

Variants affecting splicing have profound effects on protein sequence, structure, and function and are frequently found in almost all known inherited diseases^1^. Those variants that affect the highly conserved GU and AG dinucleotides of the splice acceptor and donor sites almost always result in a splicing defect, so their validation is usually unnecessary unless one needs to know the exact effects on the transcript. In contrast, variants near but outside the canonical dinucleotides are more difficult to interpret because these sites are much more variable and their effects on splicing need to be validated. According to the guidelines for the classification of disease-associated variants provided by the American College of Medical Genetics and Genomics (ACMG)^2^, such variants must be classified as variants of uncertain significance (VUS) as long as their effect has not been validated. Consequently, in a patient harboring a VUS, the cause and diagnosis of the disease have not been confirmed genetically. Therefore, the patient is considered unresolved in genetic diagnostics until the clinical significance of identified variants is validated by functional studies. Since more and more gene therapeutic approaches are under development, it is absolutely essential to confirm a diagnosis through molecular genetic approaches. Only then can patients participate in clinical trials or—if the therapeutic approach is approved—be considered for therapy. Although patient RNA should be preferred for splicing analysis, expression of the gene of interest is often restricted to cell types and tissues that are not readily accessible, as is the case for most genes associated with inherited retinal dystrophies. In addition, degradation of aberrant transcripts by nonsense-mediated mRNA decay (NMD), and the presence of normal and (various) mutant transcripts in heterozygous patients may complicate the assessment of aberrant splicing. Alternatively, minigene assays can be performed using patient genomic DNA or healthy control proband DNA into which the variant of interest has been introduced by site-directed mutagenesis. Minigene assays make use of splice reporter vectors that code for two exons with functional splice sites^3^. The intron between the exons contains a multiple cloning site in which the exon(s) of interest can be cloned. Following cloning, the resulting constructs in their wild-type and mutant versions are used to transfect eukaryotic cell lines. The transcripts obtained from the vector can then be further characterized. Since splice reporter vectors have a limited capacity of accommodating DNA fragments, most minigenes are restricted to one or few exons. This, of course, does not correspond to the natural context. However, if the wild-type construct is spliced correctly and the mutant construct shows missplicing, this can actually only be attributed to the variant that is analyzed, as the two vectors differ only with respect to the variant. Minigene assays are therefore well suited to investigate the potential of a variant in terms of missplicing.

In this study, we aimed to investigate 20 variants in the splice site regions of *CNGA3*. This gene encodes the CNGA3 subunit of the cyclic nucleotide-gated ion channel in cone photoreceptors^4^. Transcript NM_001298.3, to which the nomenclature of variants in this study refers, comprises seven coding exons (2–8) and is considered the major transcript in the human retina, but there are also isoforms lacking exon 5 or including an alternative exon spliced between exons 3 and 4^5,6^. In other tissues (kidney, heart, pineal gland, adrenal gland and testes), additional alternative splice forms were identified^7^. Biallelic mutations in *CNGA3* are the second most common cause of achromatopsia (MIM #216900), a rare congenital autosomal recessive disorder that affects cone photoreceptor function and leads to poor visual acuity, photophobia, congenital nystagmus and complete color blindness. As of July 2022, the Human Gene Mutation Database (HGMD)^8^ has listed 178 disease-causing variants in *CNGA3*. The mutation spectrum is dominated by nonsense and missense mutations (n = 163) whereas splice mutations are comparably rare (n = 7). We have recently published a mutation overview of our cohort of 889 unrelated achromatopsia patients, 304 of which harbor putatively pathogenic variants in *CNGA3*^9^. Several patients in that cohort carry splice site variants in *CNGA3* classified as VUS. Cone photoreceptors, of course, are not amenable to direct transcript analysis. In order to conclusively classify the putative splice variants we identified in our cohort of achromatopsia patients as non-pathogenic or pathogenic, we characterized them by minigene assays in the present study. To obtain a more comprehensive insight in *CNGA3*-associated splice defects, we also investigated all disease-associated *CNGA3* splice variants listed in three disease variant databases.

## Results

### Selection of variants for minigene assays

**Figure 1:**
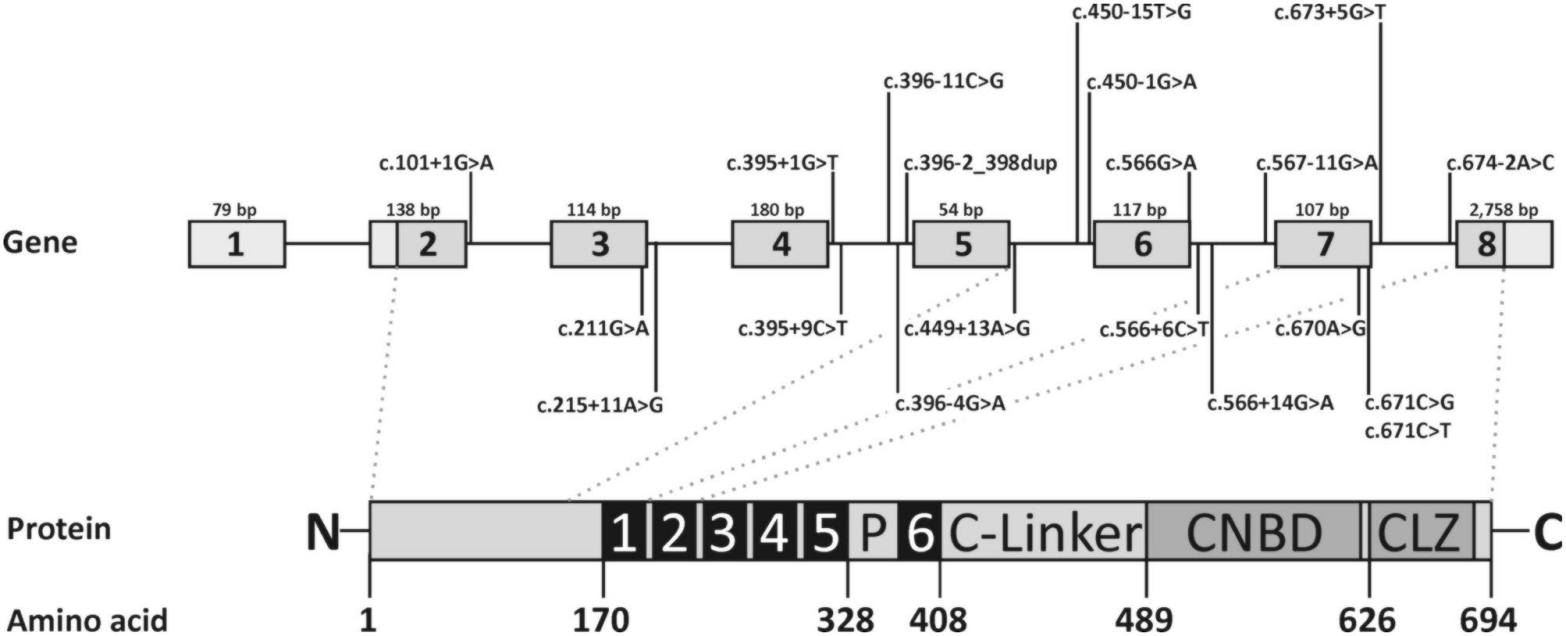
Location of splice variants in *CNGA3*. In the upper panel, the *CNGA3* gene with seven coding exons and one non-coding exon is displayed (NM_001298.3). Exons are represented by grey vertical boxes. Coding sequence is shown in a darker shade. Note that exons and intervening intronic sequence (represented by black horizontal line) are not drawn to scale. The size in bp is given above each exon. Variants for which we observed a splice defect in the splice assays are shown above the exons. Those with no effect are shown below. In the lower panel, the protein with its most important functional domains including the six transmembrane domains (in black), the pore region (P), the C-linker, the cyclic nucleotide binding domain (CNBD) and the C-terminal leucine zipper (CLZ) is shown. Positions of domains was adapted from Gofmann and colleagues^42^.

Twenty *CNGA3* sequence variants were selected to study their effects on splicing using minigene assays (Fig. 1). The variants were either identified in our cohort of achromatopsia patients^9^, or reported as disease-associated in HGMD^8^, ClinVar^10^, and LOVD^11^. Note that we have not included variants with a minor allele frequency > 0.05 in the population database gnomAD (https://gnomad.broadinstitute.org/) and that we have not considered variants deep in introns that were not predicted to create or strengthen a cryptic splice site. We also have not included the c.-37-1G>C variant in the present study as we have previously investigated this variant and described its missplicing potential^12^.

Thirteen variants were located in the consensus splice site regions (defined as the last 12 intronic to the first two exonic nucleotides of the acceptor site, and the last three exonic to the first six intronic nucleotides of the donor site, respectively), including five at the canonical positions (the last two intronic positions of the acceptor, and the first two intronic positions of the donor). Five intronic and two exonic variants were located outside the consensus splice site regions.

### In silico splicing prediction

Splice site predictions for the 20 variants analyzed in this study are given in Table 1. The three splicing prediction algorithms MaxEntScan^13^, NNSplice^14^ and SpliceAI^15^ consistently predicted no effect on splicing for eight variants, namely c.211G>A, c.215+11A>G, c.395+9C>T, c.396-4G>A, c.449+13A>G, c.566+6C>T, c.566+14G>A and c.670A>G. Only MaxEntScan predicted a subtle decrease of the donor splice site strength for variants c.671C>G and c.671C>T. For variant c.396-2_398dup only SpliceAI predicted a weak effect on splicing. For the remaining nine variants (c.101+1G>A, c.395+1G>T, c.396-11C>G, c.450-15T>G, c.450-1G>A, c.566G>A, c.567-11G>A, c.673+5G>T, and c.674-2A>C) MaxEntScan and NNSplice predicted a decrease of the splice site strength, ranging from 19 to 100% and from 11 to 100%, respectively. For the same variants, SpliceAI predicted an effect on splicing with Δscores ranging from 0.11 to 0.99.

**Table 1:** In silico analysis, observed effect in minigenes and effect on protein as determined by cDNA analysis. ^a^Negative values are upstream (5′) of the variant, and positive are downstream (3´) of the variant. Cut-off values were 0.0 for MaxEntScan and 0.0 for NNSplice. DSS, donor splice site; ASS, acceptor splice site; nt, nucleotides. ^b^Major and minor refer to the relative strength of transcripts observed on agarose gels and/or the relative ratios after subcloning.

		Splice prediction					Variant database ID		
Variant	Location	MaxEntScan % decrease of splice site strength	NNSplice % decrease of splice site strength	SpliceAI (Δ score)/pre-mRNA position^a^	Observed effect in minigene assay^b^	Deduced amino acid change	HGMD	ClinVar	LOVD
c.101+1G>A	Intron 2	− 76%	− 100%	DSS loss (0.99)/ − 1 bp	single transcript: exonic cryptic DSS is used (exon 2 lacks the last 50 nt)	p.(Val18Serfs*6)	CS1818926	VCV000208567	0000608173
c.211G>A	Exon 3	No effect	No effect	No effect	Correct splicing	p.(Ala71Thr)	–	VCV000858875	–
c.215+11A>G	Intron 3	No effect	No effect	No effect	Correct splicing	Wild-type	–	VCV000337653	0000245952
c.395+1G>T	Intron 4	− 78%	− 100%	DSS loss (0.99)/− 1 bp	Transcript: skipping of exon 4	p(.Arg72_Arg131del)	CS1818927	–	0000795727
					Transcript: skipping of exon 4 and 5	p.(Leu73_Glu150del)			
					Transcript: exonic cryptic DSS is used (exon 4 lacks the last 41 nt)	p.(Ala119Argfs*10)			
					Transcript: exonic cryptic DSS is used (exon 4 lacks the last 138 nt)	p.(Arg86_Arg131del)			
					Transcript: exonic cryptic DSS is used (exon 4 lacks the last 150 nt)	p.(Arg82_Arg131del)			
					Transcript: intronic cryptic DSS is used (the first 488 nt of intron 4 are retained)	p.(Ala133*)			
c.395+9C>T	Intron 4	No effect	No effect	No effect	Correct splicing	wild-type	–	VCV000897208	–
c.396− 11C>G	Intron 4	− 53%	− 11%	ASS loss (0.87)/ 11 bp ASS gain (0.7)/1 bp	Major transcript: skipping of exon 5	p.(Ser132_Glu150delinsArg)	CS148758	–	0,000,763,909
					Minor transcript: intronic cryptic ASS is used (the last 10 nt of intron 4 are retained)	p.(Ala133Phefs*13)			
c.396-4G>A	Intron 4	No effect	No effect	No effect	Correct splicing	wild-type	–	VCV000337656	–
c.396-2_398dup	Intron 4/exon 5	No effect	No effect	Ass gain (0.22)/ 0 bp	Single transcript: duplicated ASS site is used (the last 5 nt of intron 4 are retained)	p.(Trp134Alafs*41)	–	VCV001064481	–
c.449+13A>G	Intron 5	No effect	No effect	No effect	Correct splicing	wild-type	–	VCV000897210	–
c.450-15T>G	intron 5	− 46%	− 24%	ASS loss (0.65)/ 15 bp ASS gain (0.53)/ 1 bp	Major transcript: intronic ASS site is used (the last 14 nt of intron 5 are retained)	p.(Lys151Cysfs*27)	–	VCV001064482	–
					Minor transcript: intronic ASS site is used (the last 49 nt of intron 5 are retained)	p.(Glu150Aspfs*49)			
					Minor transcript: skipping of exon 6	p.(Lys151_Arg189del)			
					Minor transcript: correct splicing	Wild-type			
c.450- 1G>A	Intron 5	− 100%	− 100%	ASS loss (0.95)/ 1 bp	Major transcript: intronic ASS site is used (the last 154 nt of intron 5 are retained)	p.(Lys151Aspfs*24)	CS191808	VCV001453949	–
					Minor transcript: intronic ASS site is used (the last 70 nt of intron 5 are retained)	p.(Lys151Leufs*55)			
					Minor transcript: intronic ASS site is used (the last 49 nt of intron 5 are retained)	p.(Glu150Aspfs*49)			
					Minor transcript: skipping of exon 6	p.(Lys151_Arg189del)			
c.566G>A	Intron 6	− 24%	− 30%	DSS gain (0.32)/ -18 bp	Major transcript: correct splicing	p.(Arg189Lys)	–	VCV001064486	–
					Minor transcript: exonic cryptic DSS is used (exon 6 lacks the last 18 nt)	p.(Tyr184_Arg189del)			
c.566+6C>T	Intron 6	No effect	No effect	No effect	Correct splicing	Wild-type	–	VCV000337658	–
c.566+14G>A	Intron 6	No effect	No effect	No effect	Correct splicing	Wild-type	–	VCV000898384	–
c.567-11G>A	Intron 6	− 19%	− 68%	ASS loss (0.6)/ 11 bp ASS gain (0.92)/ 2 bp	Single transcript: intronic ASS is used (the last 9 nt of intron 6 are retained)	p.(Cys188_Arg189insSerPhePhe)	–	–	–
c.670A>G	Exon 7	No effect	No effect	No effect	Correct splicing	p.(Thr224Ala)	–	VCV001064489	0,000,517,243
c.671C>G	Exon 7	− 6%	No effect	No effect	Correct splicing	p.(Thr224Arg)	CM014535	–	0,000,832,034
c.671C>T	Exon 7	− 17%	No effect	No effect	Correct splicing	p.(Thr224Ile)	CM148761	–	0,000,763,923
c.673+5G>T	Exon 7	− 44%	− 30%	DSS loss (0.11)/ − 5 bp	Major transcript: correct splicing	Wild-type	CS2112742	VCV001062974	–
					Minor transcript: skipping of exon 7	p.(Ala190Phefs*10)			
c.674-2A>C	Intron 7	− 66%	− 100%	ASS loss (0.99)/ 2bp ASS loss (0.84)/ 17 bp	Major transcript: exonic cryptic ASS is used(exon 8 lacks the first 15 nt)	p.(Phe226_Gly230del)	CS148762	–	0000763995
					Minor transcript: exonic cryptic ASS is used(exon 8 lacks the first 9 nt)	p.(Gly225_Leu227del)			
					Minor transcript: correct splicing	wild-type			

Note that we did not apply thresholds to any of the algorithms and that the results of the in silico predictions were not used to exclude variants from the analysis with minigene assays, as we also aimed to evaluate whether the in silico predictions were consistent with the functional analyses.

### Minigene assays

The 20 *CNGA3* variants were tested for their effect on splicing in a pSPL3 splicing reporter minigene assay, using nine different amplicon designs. Seven minigene constructs were based on a single coding exon of *CNGA3* cloned in the HIV-tat intron which separates the two pSPL3-resident exons^16^. We also generated a minigene construct comprising two exons, namely exon 4 and exon 5. Analysis of the c.674-2A>C located in exon 8 was performed using a construct based on a hybrid exon (see Material and Methods section).

**Figure 2:**
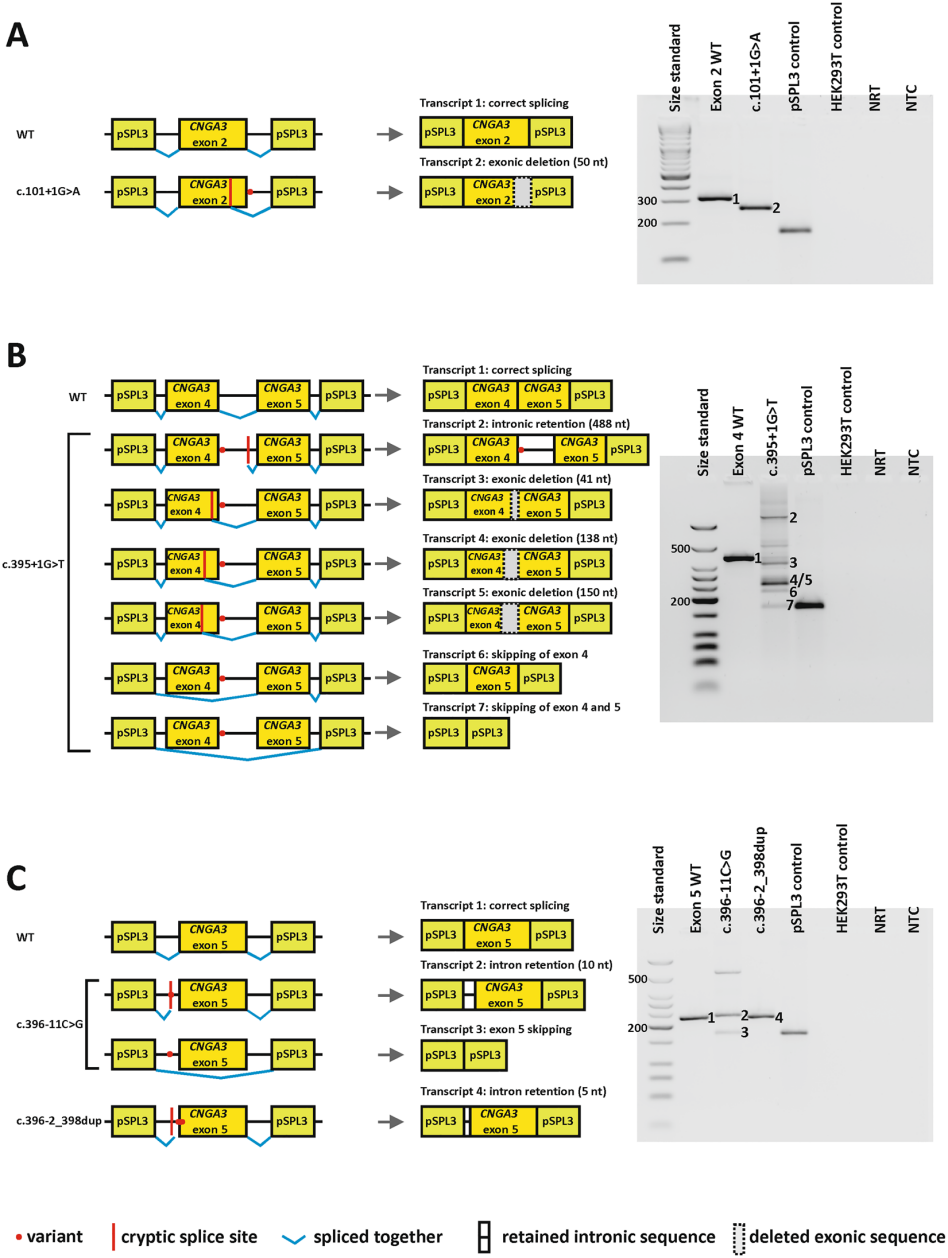
Splice defects caused by variants in minigenes comprising *CNGA3* exon 2 (**A**), exons 4–5 (**B**) and exon 5 (**C**). The left side of the composite image shows the respective minigene constructs. The location of the variants is indicated with a red dot. Cryptic splice sites are indicated by a vertical red line. pSPL3 resident exons are shown as green boxes and *CNGA3* exons are shown as yellow boxes. Intronic sequence is indicated by a black horizontal line. Observed transcripts in the splice assays are shown in the middle of the composite image. Deleted exonic sequence is indicated by a grey box with a dashed outline. Retained intronic sequence is indicated by a white box with a horizontal bar in the middle. Agarose gels (uncropped images) are shown on the far right. Loading in all gels is as follows: A size standard (low molecular weight DNA ladder, NEB or 1 kb plus ladder, NEB) is loaded in the leftmost lane, followed by the RT-PCR from transfection with the respective wild-type (WT) minigene construct. In the following lanes, RT-PCRs from transfection with the respective mutant minigene constructs were loaded, followed by the RT‐PCRs from transfection with empty pSPL3 vector, untransfected HEK293T cells, no reverse transcriptase control (NRT), and no template control (NTC). Observed transcripts are labeled with a number which is used to reference the transcripts in the middle panel. Note that not all transcripts identified by subcloning are clearly visible on the gel and, conversely, not all bands on the gel could be captured by subcloning. Sequence electropherograms showing the splice junctions of all subcloned transcripts are given in Supplementary Figure S2.

**Figure 3:**
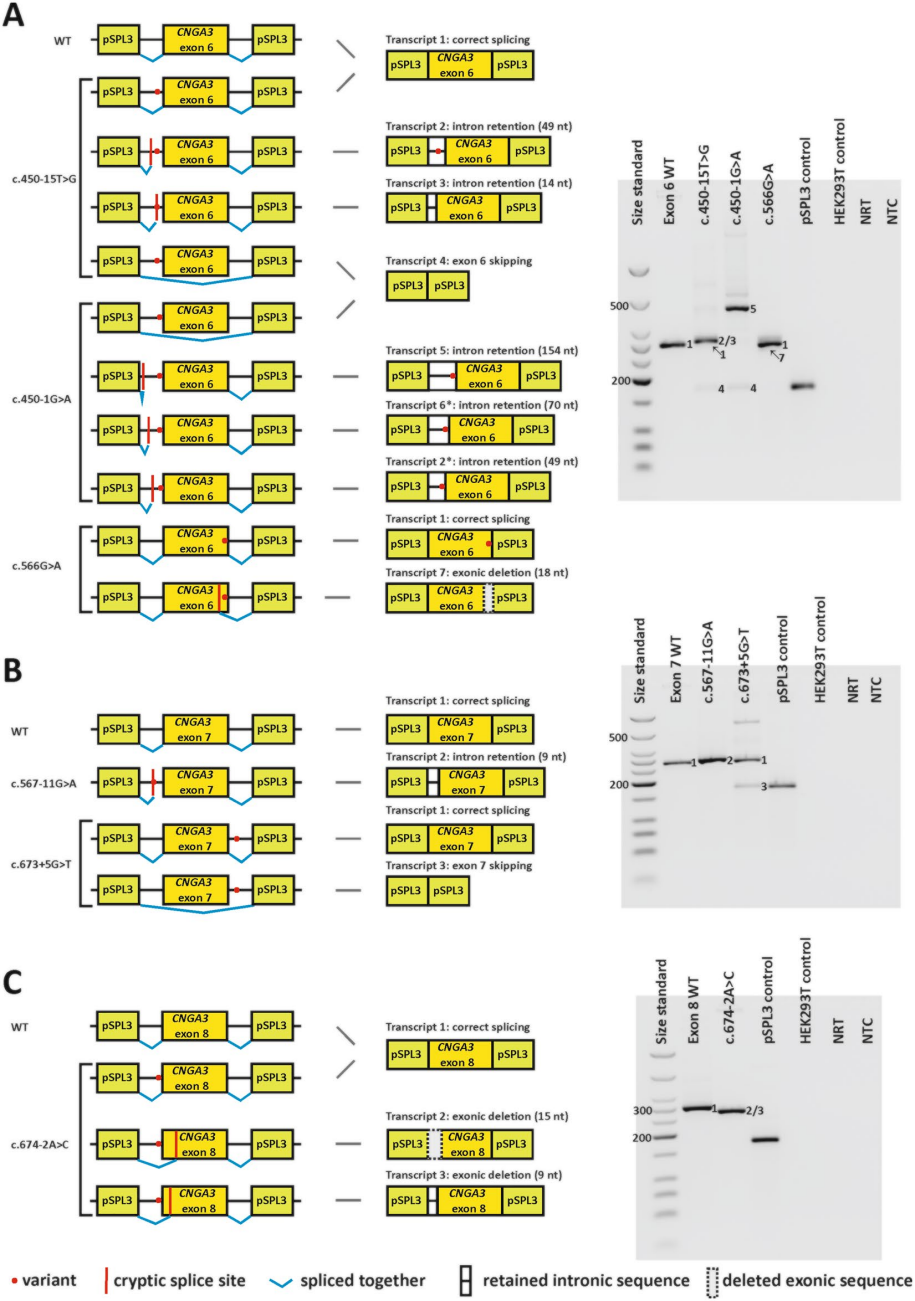
Splice defects caused by variants in minigenes comprising *CNGA3* exon 6 (**A**), exon 7 (**B**) and exon 8 (**C**). Layout and labeling correspond to that in Fig. 2. *Transcripts only detected after subcloning. Only uncropped gel images are shown.

Splice variants were studied in HEK293T cells since they are commonly used for minigene assays and no stable human cone photoreceptor cell line is yet available. In addition, we previously compared the outcome of different splice variant in HEK293T and 661W cells (a murine cell line displaying characteristics of photoreceptor cells) and found no qualitative or quantitative differences in the observed transcripts^12,17^. Following transfection of HEK293T cells, all wild-type constructs yielded a single RT-PCR product with the cloned *CNGA3* exon(s) spliced correctly between the vector-resident exons (Figs. 2, 3, Supplementary Figure S1). The 20 candidate variants were introduced into the corresponding minigene constructs by site-directed mutagenesis. Ten variants showed no effect on splicing (Supplementary Figure S1), in good agreement with the in silico splice site predictions, as all three splice algorithms consistently predicted no effect for eight of them (Table 1). Two exonic variants (c.671C>G and c.671C>T) for which only MaxEntScan had predicted a slight decrease of the splice donor strength (− 6% and − 17%), showed no splice defect in the minigene assays.

Ten variants were shown to induce different aberrant splicing events, including intronic nucleotide retention, exonic nucleotide deletion, and exon skipping (Figs. 2, 3). Overall, a splice defect was confirmed for all variants for which the three splicing algorithms consistently predicted an effect. In addition, a splice defect was observed for the variant c.396-2_398dup, for which only SpliceAI had predicted a splice defect.

Five of the variants exerting a splice defect affected the canonical positions of the splice acceptor and donor sites, namely c.101+1G>A, c.395+1G>T, c.396-2_398dup, c.450-1G>A, and c.674-2A>C. In addition, missplicing was observed for four variants affecting the consensus sequence (c.396-11C>G, c.566G>A, c.567-11G>A, and c.673+5G>T) and for one variant outside the consensus sequence (c.450-15T>G). Overall, the ten spliceogenic variants each produced one to six different transcripts. Transcripts observed as strong bands on agarose gels are referred to as "major" RT-PCR products, while weak bands or transcripts that could only be detected by subcloning are referred to as "minor" RT-PCR products.

Cryptic splice site usage was the most frequent spliceogenic mechanism (nine variants; 16 transcripts) whereas exon skipping was observed in six transcripts (five variants) (Table 1). Of note, both spliceogenic mechanisms were observed for several variants, however, never to the same extent. Eleven transcripts were predicted to introduce a premature termination codon (PTC) whereas ten transcripts were predicted to retain the reading frame. Of these, eight transcripts were predicted to lead to a deletion of several amino acid residues (ranging from three to 78 residues). In addition, one transcript was predicted to lead to an insertion of three amino acid residues, and one transcript was predicted to lead to a combined insertion/deletion of 19 amino acid residues.

In detail, variant c.101+1G>A completely destroyed the authentic donor site of exon 2. HEK293T cells transfected with this minigene construct generated a single aberrant transcript that lacked the last 50 nucleotides of exon 2, due to the use of an exonic cryptic donor site (Fig. 2A). The predicted effect on the protein is a frameshift and PTC (p.(Val18Serfs*6)).

The variant located at the canonical splice donor site of exon 4, c.395+1G>T, showed the most complex splicing pattern. Analysis of this variant was based on a minigene construct comprising exon 4 and exon 5. Subcloning and subsequent sequencing of RT-PCR products revealed six different aberrant transcripts (Fig. 2B), including skipping of exon 4, and both exon 4 and 5. Both exon skipping transcripts are predicted to result in an in frame deletion of 60 or 78 amino acids, respectively (p.(Arg72_Arg131del) and p.(Leu73_Glu150del)). In addition, several transcripts were generated that used exonic or intronic cryptic donor sites, resulting in either a frameshift and PTC (p.(Ala119Argfs*10)), deletions of 46 or 50 amino acids, respectively (p.(Arg86_Arg131del) and p.(Arg82_131del)), or a direct PTC (p.(Ala133*)).

Variant c.396-11C>G completely destroyed the authentic acceptor site of exon 5. HEK293T cells transfected with this minigene construct generated a major aberrant transcript that retained the last ten nucleotides of intron 4, due to the use of an intronic cryptic acceptor site (Fig. 2C). The predicted effect on the protein is a frameshift and PTC (p.(Ala133Phefs*13)). In addition, a minor transcript was observed that lacked exon 4 (Fig. 2C) and is predicted to lead to an in frame insertion/deletion (p.(Ser132_Glu150delinsArg)).

The second variant affecting the canonical acceptor site of exon 4, c.396-2_398dup, generated a single aberrant transcript that retained the last five nucleotides of intron 4 (Fig. 2C) and was predicted to result in a frameshift and PTC (p.(Trp134Alafs*41)).

Two variants at the acceptor site of exon 5, c.450-15T>G and c.450-1G>A, each generated different aberrant transcripts (Fig. 3A). The major transcript observed for c.450-15T>G is predicted to result in a frameshift and PTC (p.(Lys151Cysfs*27)), due to the use of an intronic cryptic acceptor site. A minor transcript for the same variant is based on the use of another intronic cryptic acceptor site further upstream that is also predicted to result in a frameshift and PTC (p.(Glu150Aspfs*49)). Two additional minor transcripts were observed for variant c.450-15T G, namely skipping of exon 6 (p.(Lys151_Arg189del)), as well as correctly spliced transcript. The variant at the canonical acceptor site of exon 6, c.450-1G>A, generated a major transcript based on the use of an intronic cryptic acceptor site that is predicted to result in a frameshift and PTC (p.(Lys151Aspfs*24)). In addition, subcloning revealed two minor transcripts that used other intronic cryptic acceptor sites further downstream, both of them predicted to result in a frameshift and PTC (p.(Lys151Leufs*55) and p.(Glu150Aspfs*49)). Another minor transcript lacked exon 6 and is predicted to result in an in frame deletion of 39 amino acids (p.(Lys151_Arg189del)).

Variant c.566G>A, located at the last nucleotide of exon 6, generated large amounts of correctly spliced transcript (Fig. 3A), which, however, is predicted to lead to an exchange of one amino acid (p.(Arg189Lys)). In addition, the variant led to the use of an exonic cryptic donor site in a minor fraction of transcripts (Fig. 3A), predicted to lead to a deletion of six amino acids (p.(Tyr184_Arg189del)).

Variant c.567-11G>A completely destroyed the authentic acceptor site of exon 7. HEK293T cells transfected with this minigene construct generated a single aberrant transcript that retained the last nine nucleotides of intron 6, due to the use of an intronic cryptic acceptor site (Fig. 3B). The predicted effect on the protein is an in frame insertion of three amino acids (p.(Cys188_Arg189insSerPhePhe)).

Variant c.673+5G>T, located at the donor site of exon 7, generated large amounts of correctly spliced transcript, but also generated a minor transcript that lacked exon 7 (Fig. 3B). The effect of exon 7 skipping is predicted as p.(Ala190Phefs*10).

Finally, variant c.674-2A>C, located at the acceptor site of the last exon, was shown to generate a major aberrant transcript that lacked the first 15 nucleotides of exon 8, due to the use of an exonic cryptic acceptor site (Fig. 3C). The predicted effect is a deletion of five amino acids (p.(Phe226_Gly230del)).

In addition, variant c.674-2A>C generated a minor transcript that lacked the first nine nucleotides of exon 8. The effect of this transcript was predicted as p.(Gly225_Leu227del). Subcloning also revealed minor amounts of correctly spliced transcript.

Note that we did not quantify the relative expression of different transcripts produced by the same variant because we do not expect the relative expression to be directly comparable to the situation in the native expressing tissue, i.e., the cone photoreceptor, as has been shown previously for other genes (e.g., *ABCA4*^18^). In addition, most ACHM-patients are compound heterozygotes, and about 2/3 of known *CNGA3* variants are missense variants. Therefore, it is very likely that CNGA3-ACHM patients with a splice variant on one allele have a missense variant on the other allele. The majority of variants were previously classified according to ACMG guidelines^2,9^, with two variants classified as benign, two as likely benign, eight as VUS, six as likely pathogenic, and two as pathogenic. We reclassified the variants considering the results of our minigene assays by applying the BS3 criterion for variants not affecting splicing and the PS3 criterion for variants that induced missplicing in the minigene assay. Of the eight VUS, two variants were reclassified as likely benign and four as likely pathogenic, while two remained unchanged. Two variants were reclassified from likely pathogenic to pathogenic. For the remaining likely benign as well as the likely pathogenic variants, the ACMG classification was confirmed (Table 2).

**Table 2:** Variant classification.

	ACMG classification	
Variant	Prior minigene assays	After functional validation by minigene assays
c.101+1G>A	**Pathogenic** PS1_Very strong, PM2_Moderate, PP5_Supporting, PP3_supporting	**Pathogenic** PS1_Very strong, PM2_Moderate, PP5_Supporting, PP3_supporting, PS3_Strong
c.211G>A/p.(Ala71Thr)	**VUS** PM2_Moderate, PP2_Supporting, BP4_supporting	**Likely benign** PM2_Moderate, PP2_Supporting, BP4_supporting, BS3_Strong
c.215+11A>G	**Benign** BS1_Strong, BS2_Strong, BP7_Supporting, BP6_Strong	**Benign** BS1_Strong, BS2_Strong, BP7_Supporting, BP6_Strong, BS3_Strong
c.395+1G>T	**Likely pathogenic** PVS1_Strong, PM2_Moderate, PP3_supporting	**Pathogenic** PVS1_Strong, PM2_Moderate, PP3_supporting, PS3_Strong
c.395+9C>T	**VUS** PM2_Moderate	**VUS** PM2_Moderate, BS3_Strong
c.396-11C>G	**VUS** PP1_Supporting, PM2_Moderate, PP3_supporting	**Likely pathogenic** PP1_Supporting, PM2_Moderate, PP3_supporting, PS3_Strong
c.396-4G>A	**Benign** BS1_Strong, BP4_Supporting, BP6_Strong	**Benign** BS1_Strong, BP4_Supporting, BP6_Strong, BS3_Strong
c.396-2_398dup	**Likely pathogenic** PM2_Moderate, BP4_supporting, PVS1_very strong	**Pathogenic** PM2_Moderate, BP4_supporting, PVS1_very strong, PS3_Strong
c.449+13A>G	**VUS** PM2_Moderate, BP7_Supporting	**Likely benign** PM2_Moderate, BP7_Supporting, BS3_Strong
c.450-15 T>G	**VUS** PM2_Moderate, PP3_supporting	**Likely pathogenic** PM2_Moderate, PP3_supporting, PS3_Strong
c.450-1G>A	**Likely pathogenic** PVS1_Strong, PM2_Moderate, PP3_supporting	**Pathogenic** PVS1_Strong, PM2_Moderate, PP3_supporting, PS3_Strong
c.566G>A/p.(Arg189Lys)	**VUS** PM2_Moderate, PM1_Supporting, PP2_Supporting, PP3_Supporting	**Likely pathogenic** PM2_Moderate, PM1_Supporting, PP2_Supporting, PP3_Supporting, PS3_Supporting
c.566+6C>T	**Likely benign** PM2_Moderate, BP4_Supporting, BP6_Supporting	**Likely benign** PM2_Moderate, BP4_Supporting, BP6_Supporting, BS3_Strong
c.566+14G>A	**Likely benign** PM2_Moderate, BP7_Supporting, BP6_Supporting	**Likely benign** PM2_Moderate, BP7_Supporting, BP6_Supporting, BS3_Strong
c.567-11G>A	**VUS** PM2_Moderate	**Likely pathogenic** PM2_Moderate, PS3_Strong
c.670A>G/p.(Thr224Ala)	**Likely pathogenic** PP3_Strong, PM2_Moderate, PP2_Supporting, PM5_supporting	**Likely pathogenic** PP3_Strong, PM2_Moderate, PP2_Supporting, PM5_supporting, BS3_Strong
c.671C>G/p.(Thr224Arg)	**Pathogenic** PP3_Strong, PM2_Moderate, PP2_Supporting, PP1_supporting, PS3_strong	**Pathogenic** PP3_Strong, PM2_Moderate, PP2_Supporting, PP1_supporting, PS3_strong,
c.671C>T/p.(Thr224Ile)	**Likely pathogenic** PP3_Strong, PM2_Moderate, PP2_Supporting, PM5_supporting	**Likely pathogenic** PP3_Strong, PM2_Moderate, PP2_Supporting, PM5_supporting, BS3_Strong
c.673+5G>T	**VUS** PM2_Moderate, PP3_Supporting	**VUS** PM2_Moderate, PP3_Supporting, PS3_Supporting
c.674-2A>C	**Likely pathogenic** PVS1_Strong, PM2_Moderate; PP3_supporting	**Pathogenic** PVS1_Strong, PM2_Moderate; PP3_supporting, PS3_Strong

Variant classification was performed using the classification tool from Franklin (https://franklin.genoox.com). Variants whose classification has changed after the minigene assays are highlighted. Experimental evidence of a splice defect was evaluated with the ACMG evidence criterion PS3_strong and exclusion of one with BS3_strong. In the case of the hypomorphic variants c.673+5G>T and c.566G>A, the PS3_supporting criterion was chosen. The variant c.671C>T/p.(Thr224Arg) has been shown to impair CNG channel function and therefore, the PS3_strong criterion was applied despite the minigene assay results. For a description of all evidence criteria please refer to ACMG guidelines^2^.

## Discussion

The most straightforward approach to validate splicing defects is based on direct mRNA analysis in patient-derived cells. The *CNGA3* gene encodes the main subunit of the cyclic nucleotide-gated ion channel in cone photoreceptors^4^. These cells are, of course, not accessible for transcript analysis. Therefore, the functional analysis of 20 putative splice site variants in the *CNGA3* gene was performed by minigene assays in our study. It is important to note that minigene assays can only determine the potential of a variant to cause missplicing. The exact outcome in the affected cell type might be different. However, several studies that focused on the comparison between minigene assays and patient RNA analyses observed a high concordance between the two methods^19–21^. Occasionally, the lack of genetic context in minigene assays and the choice of the heterologous cell system can lead to non-concordant results^22–25^.

Prior to the minigene assays, the 20 variants were evaluated using three in silico splice prediction algorithms. The minigene assays were performed for all variants, whether or not an effect on splicing was predicted in silico as we wanted to establish an unbiased approach to test whether both approaches were consistent. On the whole, the concordance between both approaches was very high. The splicing defect of nine variants that showed an effect in the minigene assays was consistently predicted by all three algorithms, but the effect of one variant (c.396-2_398dup) was predicted only by SpliceAI. It has been repeatedly observed that this deep neural network outperforms other splice prediction algorithms^26,27^. In fact, in our study, we have observed a concordance rate of 100% between the predictions of SpliceAI and the minigene assays. For NNSplice and MaxEntScan this rate was 95% and 85%, respectively.

Alternative splice site usage was the most frequent spliceogenic mechanism we observed (15 transcripts from nine variants) while exon skipping occurred in six aberrant transcripts from five variants. It has been proposed that exon skipping is the preferred pathway when the immediate vicinity of the affected exon–intron junction is devoid of alternative cryptic splice sites^28^. Of note, the number of cryptic splice sites does not increase linearly with the size of the intronic regions: Dawes and colleagues^29^ have recently shown that 87% of cryptic splice donor sites lie within 250 nucleotides of the authentic donor site. With one exception, our minigene constructs always included more than 250 nucleotides downstream of the annotated donor. Had we cloned shorter flanking intronic sequences, we might have observed exon skipping more frequently, but this might not have reflected the spliceogenic mechanism in vivo.

For four variants (c.395+1G>T, c.396-11C>G, c.450-15T>G, and c.450-1G>A) we observed that both spliceogenic mechanisms (alternative splice site usage and exon skipping) were used in parallel. Indeed, it has been repeatedly observed that the same splice site variant can activate different spliceogenic mechanisms, both in vitro and *in vivo*^21,30–32^. The different spliceogenic mechanisms have different consequences on the amino acid level. For instance, variant c.396-11C>G causes skipping of exon 5 leading to an in frame insertion/deletion (p.(Ser132_Glu150delinsArg)) as well as to intron retention leading to a frameshift and PTC (p.(Ala133Phefs*13)). All frameshift or PTC variants observed can be considered functional null alleles since they result in the elimination of most, if not all, functional domains of the CNGA3 subunit (Fig. 1). For variants c.395+1G>T, c.396-11C>G, c.450-15 T>G and c.450-1G>A multiple misspliced transcripts were detected that are predicted to cause in frame deletions of various size (19 to 60 amino acids). These comprise either the N-terminus or parts of the N-terminus and the first transmembrane domain. Regions in the N-terminus of CNGA3 are involved in binding of phosphoinositides and can—via intersubunit interactions with the C-terminus—control phosphoinositide-dependent channel activity^33^. Large in frame deletions might hamper this mechanism, but experimental studies are needed to prove this assumption.

For variants c.566G>A, c.567-11G>A and c.674-2A>C small in frame deletions or insertions spanning three to five amino acids were observed which are located within transmembrane domain (TD) 1 or the extracellular loop connecting TD1 and TD2 and are thus most likely to impair channel folding and hereby functionality. However, a conclusive assessment of these variants is only possible with the aid of a functional assay.

While the minigene assays of 18 variants in our study were very straightforward, the analysis of two variants was more challenging. Variant c.395+1G>T, which affects the splice donor site of exon 4, was first analyzed in a minigene construct that only comprised *CNGA3* exon 4. However, with this minigene construct, we observed multiple transcripts for variant c.395+1G>T that were generated by the use of several cryptic donor sites within the pSPL3 tat intron (data not shown). The use of pSPL3 cryptic splice donor sites has been observed previously, leading to the design of a modified vector, pSPL3B, that lacks the corresponding part of the tat intron^34^. Instead of using pSPL3B, we analyzed the c.395+1G>T variant in a minigene construct containing both *CNGA3* exon 4 and exon 5 to obtain a more authentic picture of the spliceogenic mechanism. Indeed, we no longer observed the use of vector specific cryptic donor splice sites with this construct. Instead, we observed exon skipping of both exon 4 alone, or exon 4 and exon 5 together, as well as the use of cryptic donor sites either in exon 4 or intron 4. Ideally, heterologous splice assay should be performed in a construct containing the entire genomic region of a gene. Of course, this is hardly possible because eukaryotic introns often comprise several kilobases of sequence, which precludes cloning in a conventional bacterial plasmid.

The second problem we faced was the analysis of the c.674-2A>C variant. Exon trapping vectors such as pSPL3, in which the exons of interest are cloned between two vector-resident exons, are only suitable for the splice analysis of internal exons. Variant c.674-2A>C, however, is located at the acceptor splice site of exon 8. Exon 8 is the last exon in transcript NM_001298.3, consequently it lacks a natural donor splice site. To be able to study the effect of the c.674-2A>C variant with a pSPL3-based minigene assay, we generated a hybrid minigene construct. Using overlap extension PCR, we fused a fragment comprising the last 378 nucleotides of intron 7 and the first 122 nucleotides of exon 8 with a fragment comprising the first 284 nucleotides of intron 7. In this way, we obtained a truncated version of exon 8 with a functional donor splice site. The wild-type construct generated a transcript with the truncated *CNGA3* exon spliced correctly between the vector-resident exons, thereby showing that the truncated exon indeed had two functional splice sites that were recognized by the spliceosome. The mutant construct yielded a major transcript that made use of an exonic cryptic acceptor site. This most likely also corresponds to the situation in vivo, since the last exon of a transcript cannot be skipped. Whether the same exonic cryptic acceptor site is used in vivo or a site further downstream (which is not part of our minigene construct) remains to be determined. In addition, we observed a minor transcript that made use of an intronic cryptic acceptor site as well as a minor fraction of correctly spliced transcripts.

Variants c.566G>A and c.673+5G>T showed a splicing defect but also generated large amounts of correctly spliced transcript. Whether this is also the case in the native expressing cone photoreceptor remains elusive. The exonic c.566G>A variant is predicted to cause an amino acid exchange (p.(Arg189Lys)) located at the extracellular loop between TD1 and TD2. It remains to be determined whether this variant impairs CNG channel function, thereby still exerting a pathogenic effect. In contrast, the intronic c.673+5G>T variant that leads to exon skipping in a minor fraction of transcripts must be considered hypomorphic. It may be un-masked as being deleterious in homozygous state or in compound heterozygous state with another pathogenic allele. In our cohort of achromatopsia patients, we have not observed the c.673+5G>T variant yet. So far, it was only observed once in single heterozygous state in a patient diagnosed with retinitis pigmentosa^35^. This is most likely an incidental finding, as *CNGA3* is thought to be primarily involved in cone dysfunction disorders^36^. At present, it is not possible to determine whether the c.673+5G>T variant can cause a phenotype of achromatopsia or related disorders in a biallelic state.

The results of our minigene assays did not change the ACMG classification of ten variants, thus confirming their previous classification. In contrast, four variants could be upgraded from likely pathogenic to pathogenic (c.395+1G>T, c.396-2_398dup, c.450-1G>A, and c.674-2A>C). More importantly, four variants could be upgraded from VUS to likely pathogenic (c.396-11C>G, c.450-15 T>G, c.566G>A and c.567-11G>A) and two variant could be downgraded from VUS to likely benign (c.211G>A and c.449+13A>G). However, it is important to state that four of the variants that did not show a splicing defect might exert a pathogenic effect because they are predicted to lead to an amino acid exchange, namely c.211G>A; p.(Ala71Thr), c.670A>G; p.(Thr224Ala), c.671C>G; p.(Thr224Arg), and c.671C>T; p.(Thr224Ile). Indeed, the variant c.671C>G; p.(Thr224Arg) has been shown to completely abolish CNG channel function in heterologous expression systems^37,38^. Further functional studies are needed to determine whether the three remaining variants act similarly by impacting channel function.

To conclude, we have tested 20 *CNGA3* splice-site variants in an in vitro splice assay based on the pSPL3 vector. This approach has proven to be a straightforward and appropriate strategy to characterize the spliceogenic mechanisms of putative splice variants. The subsequent application of the ACMG evidence criteria PS3/BS3 enabled re-classification of 75% of the VUS into either likely benign or likely pathogenic, thereby improving genetic diagnostics in achromatopsia patients.

## Methods

### Nomenclature

Variant nomenclature in this study is in accordance with Human Genome Variation Society recommendations^39^ and based on GenBank accession numbers NM_001298.3 and NP_001289.1, respectively. The consequence of variants on the protein level was assessed using Mutalyzer (https://mutalyzer.nl/).

### Variant classification

Variant classification in this manuscript was performed using the classification tool from Franklin (https://franklin.genoox.com) which is based on ACMG guidelines^2^. The following classification categories were used: pathogenic, likely pathogenic, variant of uncertain significance, likely benign and benign.

### In silico splice predictions

The prediction of splicing alterations was performed with MaxEntScan^13^ (for 5′ splice sites: http://hollywood.mit.edu/burgelab/maxent/Xmaxentscan_scoreseq.html and for 3′ splice sites: http://hollywood.mit.edu/burgelab/maxent/Xmaxentscan_scoreseq_acc.html) using the maximum entropy model, NNSplice^14^ (https://www.fruitfly.org/seq_tools/splice.html) with thresholds set to zero and the SpliceAI^15^ lookup tool from the Broad Institute (https://spliceailookup.broadinstitute.org/, accessed on 15 April 2022) applying default settings.

### Minigene assays

Minigene assays were based on the pSPL3 exon trapping vector as described previously^40^. Cloned inserts included one or two exons of *CNGA3* with a variable length of flanking 5′ and 3′ intronic sequences (primer sequences are given in Supplementary Table S1). Analysis of the c.674-2A>C required the generation of a hybrid exon. Using overlap extension PCR, a fragment comprising the last 378 nucleotides of intron 7 and the first 122 nucleotides of exon 8 was fused with a fragment comprising the first 284 nucleotides of intron 7 in order to obtain an exon with a functional splice donor site (see Supplementary Table S1).

Genomic DNA from a healthy control individual was amplified using a proofreading DNA polymerase and cloned into the pSPL3 minigene plasmid vector. After verifying that the wild-type minigene constructs generated correctly spliced RNA, 20 variants were introduced into the wild-type constructs by site-directed mutagenesis. Mutagenesis primers are given in Table S2 and were designed following the guidelines of the QuikChange® Site-Directed Mutagenesis Kit (Stratagene, La Jolla, CA, United States) using the PrimerX online tool (https://www.bioinformatics.org/primerx/index.htm). Mutagenesis was performed as described previously^41^. Minigenes were verified by Sanger sequencing of all exons as well as flanking intronic sequences following standard protocols using the SupreDye™ v1.1 Cycle Sequencing Kit (AdvancedSeq LLC, CA, USA). The resulting minigene constructs in their wild-type and mutant versions were used to transfect HEK293T/17 cells (ATCC® CRL-11268™), which were then analyzed with respect to splicing of minigene-derived transcripts using reverse transcription polymerase chain reaction (RT-PCR). The cDNA synthesis and RT-PCRs were performed in duplicate. Subcloning of RT-PCR products was performed using the CloneJET PCR Cloning Kit (Thermo Fisher Scientific, Dreieich, Germany) according to manufacturer´s instructions.

## Data availability

The datasets generated and/or analysed during the current study are available from the corresponding author on reasonable request. The novel variant identified in this study (c.567-11G>A) was submitted to the Global Variome shared LOVD database and can be accessed using the following URL: https://databases.lovd.nl/shared/variants/CNGA3?search_position_c_start=567&search_position_c_start_intron=-11&search_position_c_end=567&search_position_c_end_intron=-11&search_vot_clean_dna_change=%3D%22c.567-11G%3EA%22&search_transcriptid=00005380 under the variant ID 0000874006. All other variants described in this study have been previously deposited by us and others in HGMD, ClinVar or LOVD (see accession numbers in Table 1).

## Supplementary Information

Supplementary Information.
