# Supplementary material for: Systematic analysis of CNGA3 splice variants identifies different mechanisms of aberrant splicing
Source: Sci Rep. 2023 Feb 18;13:2896. doi: 10.1038/s41598-023-29452-9 (PMC9938885; doi:10.1038/s41598-023-29452-9)
Supplement: Supplementary file 1 — Supplementary Information. [file 41598_2023_29452_MOESM1_ESM.pdf]

Supplementary Data

Title: Systematic analysis of *CNGA3* splice variants identifies different mechanisms of aberrant splicing

Authors: Peggy Reuter, Magdalena Walter, Susanne Kohl, and Nicole Weisschuh

Supplementary Table S1: Primers for wildtype minigene constructs

<table>
<tr><th>Primer name</th><th>Sequence (5´→3´)</th><th colspan="2">Amplicon size (bp)</th><th>Flanking intronic region (upstream/ downstream bp)</th></tr>
<tr><td>CNGA3-exon 2-NotI</td><td>TATATATAGCGGCCGCGGCCCTTCATTTATTTATTCATTCACT</td><td colspan="2" rowspan="2">700</td><td rowspan="2">287/275</td></tr>
<tr><td>CNGA3-exon 2-BamHI</td><td>TATATATAGGATCCGCTGCATTTGAGTCTTCATTTTCT</td></tr>
<tr><td>CNGA3-exon 3-NotI</td><td>TATATATAGCGGCCGCGCCAGGCCAGCATGTACT</td><td colspan="2" rowspan="2">693</td><td rowspan="2">269/310</td></tr>
<tr><td>CNGA3-exon 3-BamHI</td><td>TATATATAGGATCCTGGCTGCTCTTACCACCTTC</td></tr>
<tr><td>CNGA3-exon 4-NotI</td><td>TATATATAGCGGCCGCCCTGCACTTGGCAGTCATAA</td><td colspan="2" rowspan="2">850</td><td rowspan="2">379/291</td></tr>
<tr><td>CNGA3-exon 4-BamHI</td><td>TATATATAGGATCCGATTTCCAGGGGACCCTAAC</td></tr>
<tr><td>CNGA3-exon 5-NotI</td><td>TATATATATAGCGGCCGCGTAACTAATCACAAAGCATTTTGG</td><td colspan="2" rowspan="2">487</td><td rowspan="2">183/250</td></tr>
<tr><td>CNGA3-exon 5-BamHI</td><td>TATATATATAGGATCCCTTTATGGATGAGAAAAATGAGAG</td></tr>
<tr><td>CNGA3-exon 4-5-NotI</td><td>TATATATAGCGGCCGCCCTGCACTTGGCAGTCATAA</td><td colspan="2" rowspan="2">3834</td><td rowspan="2">379/188</td></tr>
<tr><td>CNGA3-exon 4-5-BamHI</td><td>TATATATAGGATCCATCTCATCCCTCCCCGACTT</td></tr>
<tr><td>CNGA3-exon 6-NotI</td><td>TATATATAGCGGCCGCAAGGGTGTGGATGTTGTCGT</td><td colspan="2" rowspan="2">687</td><td rowspan="2">291/279</td></tr>
<tr><td>CNGA3-exon 6-BamHI</td><td>TATATATAGGATCCCCTGCATCCTCCTCGTACA</td></tr>
<tr><td>CNGA3-exon 7-NotI</td><td>TATATATAGCGGCCGCCCTGGAAAACGGTACCTCCT</td><td colspan="2" rowspan="2">686</td><td rowspan="2">295/284</td></tr>
<tr><td>CNGA3-exon 7-BamHI</td><td>TATATATAGGATCCCAAGAACAAGGCCCAGAGAG</td></tr>
<tr><td colspan="5">Hybrid construct for exon 8</td></tr>
<tr><td>CNGA3-exon 8-NotI</td><td>TATATATAGCGGCCGCGGCCCTCTGTCACTTTTTGA</td><td rowspan="2">284</td><td rowspan="4">784</td><td rowspan="4">378/284</td></tr>
<tr><td>Overlap extension reverse</td><td>cctggggcacactcacCTTTAAGTAAGCCAGGTCGGT</td></tr>
<tr><td>Overlap extension forward</td><td>ACCGACCTGGCTTACTTAAAGgtgagtgtgccccagg</td><td rowspan="2">500</td></tr>
<tr><td>CNGA3-exon 7-BamHI</td><td>TATATATAGGATCCCAAGAACAAGGCCCAGAGAG</td></tr>
</table>

Binding sites for restriction enzymes are indicated in italics. 5´-extension tails to the restriction sites are indicated in bold.

Supplementary Table S2: Primers used for site-directed mutagenesis

**Primer name**	**Sequence (5´→3´)**
*CNGA3*_IVMc.101+1G>A_F	GAAAATGGCCTCAGCAG**a**taagatgggctaagatgg
*CNGA3*_IVMc.101+1G>A_R	ccatcttagcccatctta**t**CTGCTGAGGCCATTTTC
*CNGA3*_IVMc.211G>A_F	GGCCAGGGGATC**A**CCAGgtaactgacc
*CNGA3*_IVMc.211G>A_R	ggtcagttacCTGG**T**GATCCCCTGGCC
*CNGA3*_IVMc.215+11A>G_F	CAGgtaactgacc**g**gcctcagtccctac
*CNGA3*_IVMc.215+11A>G_R	gtagggactgaggc**c**ggtcagttacCTG
*CNGA3*_IVMc.395+1G>T_F	GCAGACAGAGGGAGAAG**t**taaggaacggaaaag
*CNGA3*_IVMc.395+1G>T_R	cttttccgttcctta**a**CTTCTCCCTCTGTCTGC
*CNGA3*_IVMc.395+9C>T_F	GGGAGAAGgtaaggaa**t**ggaaaagaagaaggggc
*CNGA3*_IVMc.395+9C>T_R	gccccttcttcttttcc**a**ttccttacCTTCTCCC
*CNGA3*_IVMc.396-11C>G_F	gatgttctctcta**g**cttcccgcagCGCC
*CNGA3*_IVMc.396-11C>G_R	GGCGctgcgggaag**c**tagagagaacatc
*CNGA3*_IVMc.396-2_398dup_F	ccttcccgc**agCGC**agCGCCTGGC
*CNGA3*_IVMc.396-2_398dup_R	GCCAGGCGct**GCGct**gcgggaagg
*CNGA3*_IVMc.396-4G>A_F	ctctaccttccc**a**cagCGCCTGGCC
*CNGA3*_IVMc.396-4G>A_R	GGCCAGGCGctg**t**gggaaggtagag
*CNGA3*_IVMc.449+13A>G_F	gtaagtacccac**g**cacccagcagagcc
*CNGA3*_IVMc.449+13A>G_R	ggctctgctgggtg**c**gtgggtacttac
*CNGA3*_IVMc.450-15T>G_F	gcgctgtttgtgta**g**gtgtgggtttccagG
*CNGA3*_IVMc.450-15T>G_R	Cctggaaacccacac**c**tacacaaacagcgc
*CNGA3*_IVMc.450-1G>A_F	gtatgtgtgggtttcca**a**GAAGAAGACGAAAAAGAAGG
*CNGA3*_IVMc.450-1G>A_R	CCTTCTTTTTCGTCTTCTTC**t**tggaaacccacacatac
*CNGA3*_IVMc.566+14G>A_F	gtaagcgacaggg**a**tggaaggtgcagcg
*CNGA3*_IVMc.566+14G>A_R	cgctgcaccttcca**t**ccctgtcgcttac
*CNGA3*_IVMc.566+6C>T_F	CTTATTTGCAGgtaag**t**gacaggggtggaaggtg
*CNGA3*_IVMc.566+6C>T_F	caccttccacccctgtc**a**cttacCTGCAAATAAG
*CNGA3*_IVMc.566G>A_F	GTATCTGCTTATTTGCA**A**gtaagcgacaggggtgg
*CNGA3*_IVMc.566G>A_R	ccacccctgtcgcttac**T**TGCAAATAAGCAGATAC
*CNGA3*_IVMc.567-11G>A	ccatctcccacat**a**gcttctttagGGCC
*CNGA3*_IVMc.567-11G>A	GGCCctaaagaagc**t**atgtgggagatgg
*CNGA3*_IVMc.670A>G_F	GTACGAGCTCGG**G**CAGgtgagtgtgc
*CNGA3*_IVMc.670A>G_R	gcacactcacCTG**C**CCGAGCTCGTAC
*CNGA3*_IVMc.671C>G_F	GTACGAGCTCGGA**G**AGgtgagtgtgcc
*CNGA3*_IVMc.671C>G_R	ggcacactcacCT**C**TCCGAGCTCGTAC
*CNGA3*_IVMc.671C>T_F	GTACGAGCTCGGA**T**AGgtgagtgtgccc
*CNGA3*_IVMc.671C>T_R	gggcacactcacCT**A**TCCGAGCTCGTAC
*CNGA3*_IVMc.673+5G>T_F	CGGACAGgtga**t**tgtgccccaggcc
*CNGA3*_IVMc.673+5G>T_R	ggcctggggcaca**a**tcacCTGTCCG
*CNGA3*_IVMc.674-2A>C_F	cctccatcttcttcttt**c**gGTTTTCTCGAGCAAG
*CNGA3*_IVMc.674-2A>C_R	CTTGCTCGAGAAAACc**g**aaagaagaagatggagg

Intronic nucleotides are indicated in lowercase letters, exonic nucleotides in capital letters. The introduced nucleotides are indicated in bold. F, forward; R, reverse.

Supplementary Figure S1

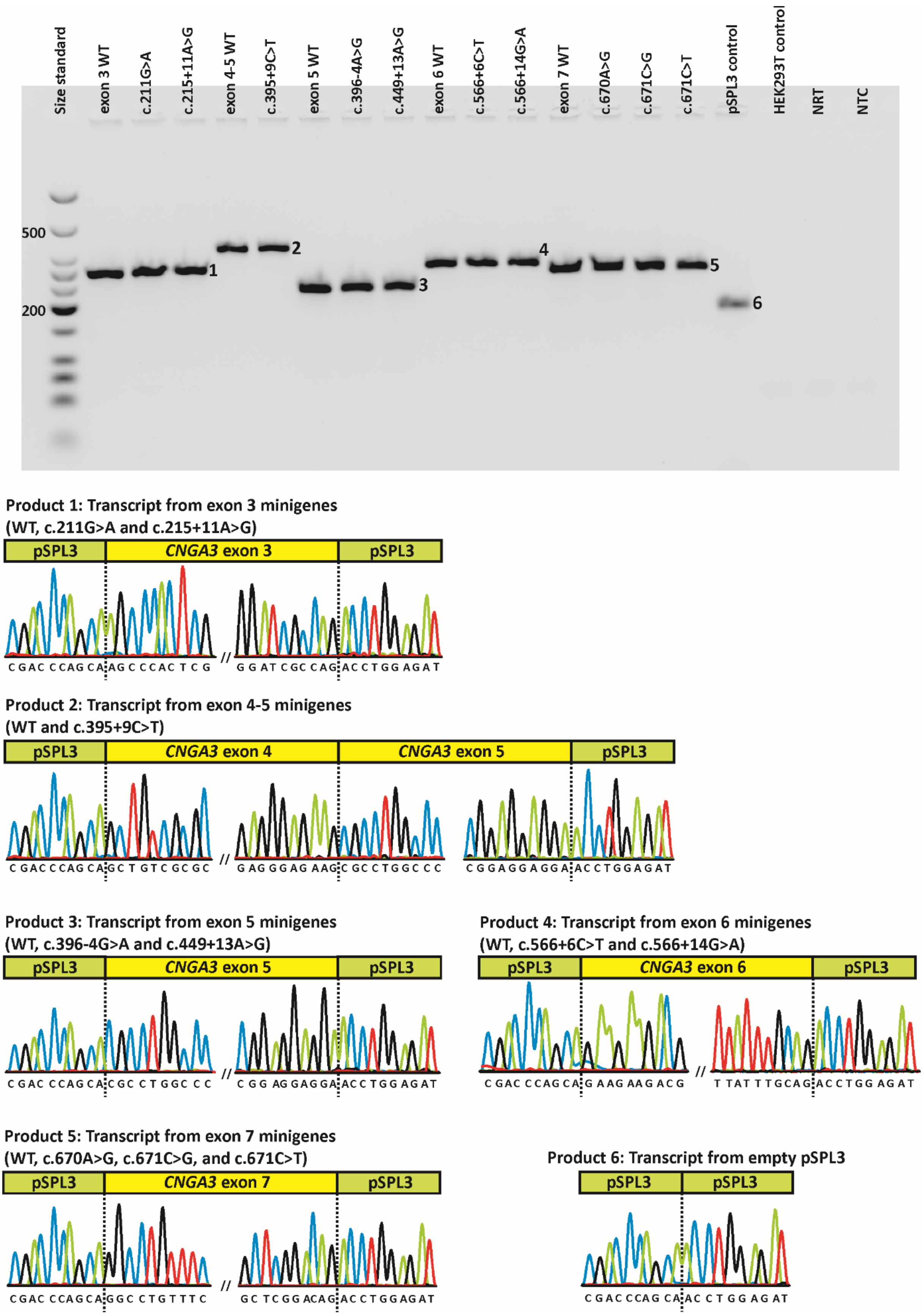

Supplementary Figure S2

Figure 2A transcript 1: Correct splicing (exon 2 wild-type minigene)

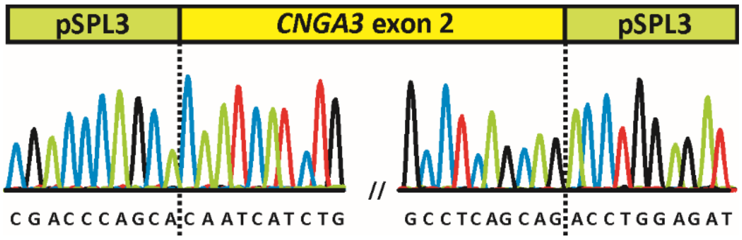

Figure 2A transcript 2: Deletion of the last 15 nucleotides of exon 2 (c.101+1G>A minigene)

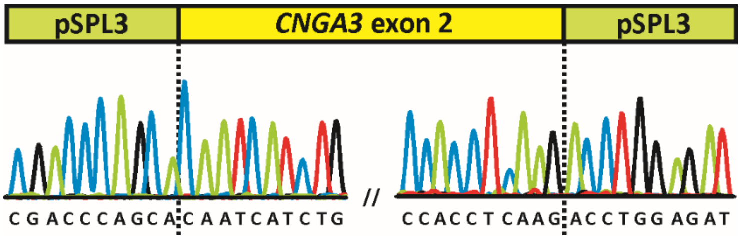

Figure 2B transcript 1: Correct splicing (exon 4-5 wild-type minigene)

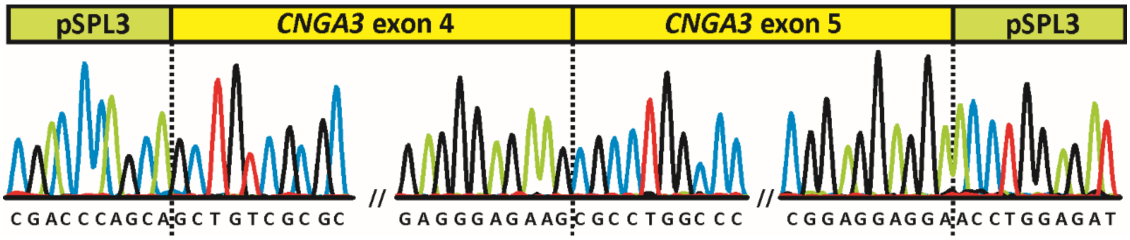

Figure 2B transcript 2: Retention of the first 488 nucleotides of intron 4 (c.395+1G>A minigene)

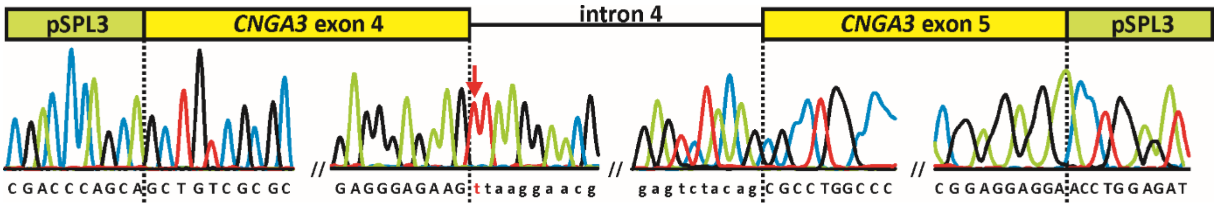

Figure 2B transcript 3: Deletion of the last 41 nucleotides of exon 4 (c.395+1G>A minigene)

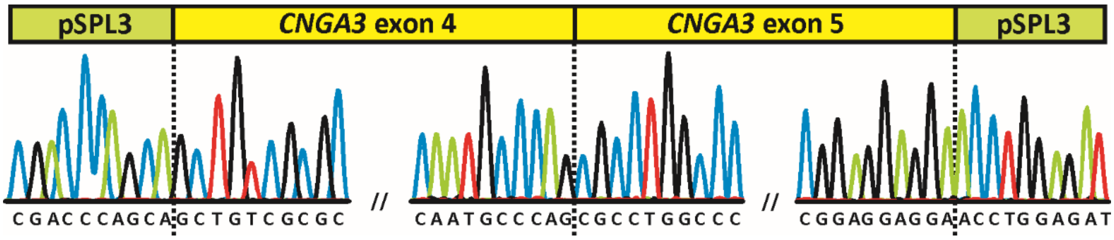

Figure 2B transcript 4: Deletion of the last 138 nucleotides of exon 4 (c.395+1G>A minigene)

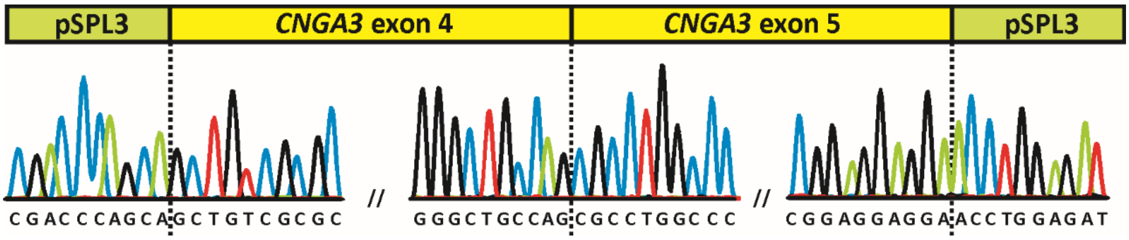

Figure 2B transcript 5: Deletion of the last 150 nucleotides of exon 4 (c.395+1G>A minigene)

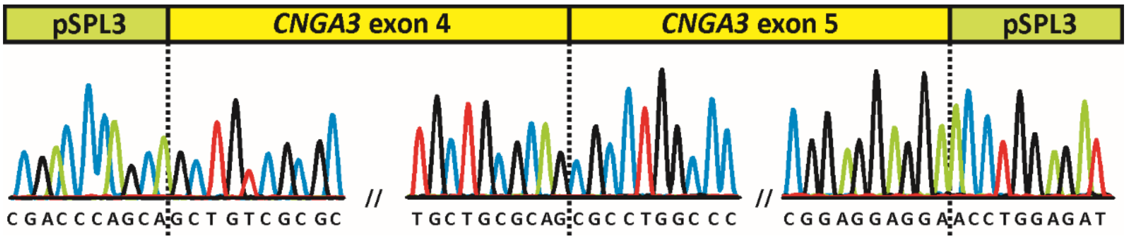

Figure 2B transcript 6: Skipping of exon 4 (c.395+1G>A minigene)

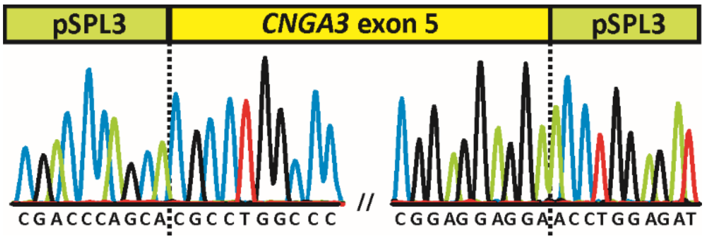

Figure 2B transcript 7: Skipping of exon 4 and exon 5 (c.395+1G>A minigene)

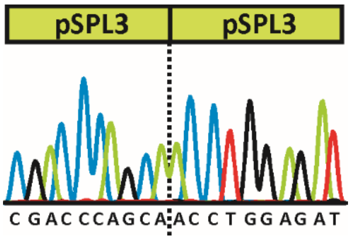

Figure 2C transcript 1: Correct splicing (exon 5 wild-type minigene)

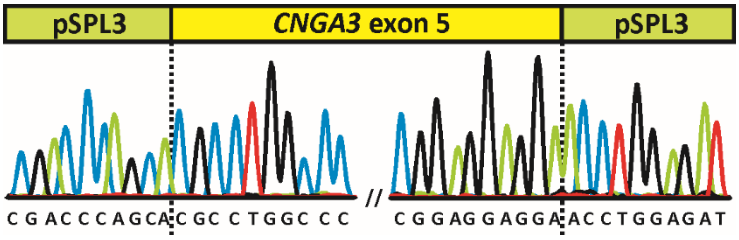

Figure 2C transcript 2: Retention of the last 10 nucleotides of intron 4 (c.396-11C>G minigene)

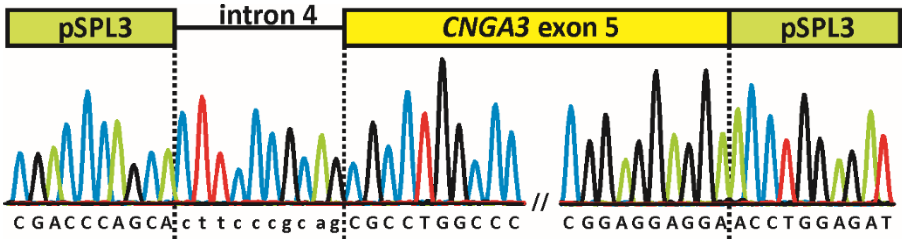

Figure 2C transcript 3: Skipping of exon 5 (c.396-11C>G minigene)

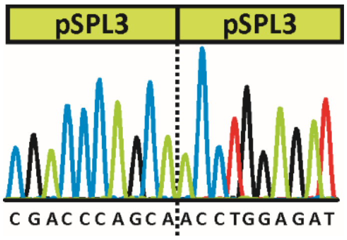

Figure 2C transcript 4: Retention of the last 4 nucleotides of intron 4 (c.396-2_398dup minigene)

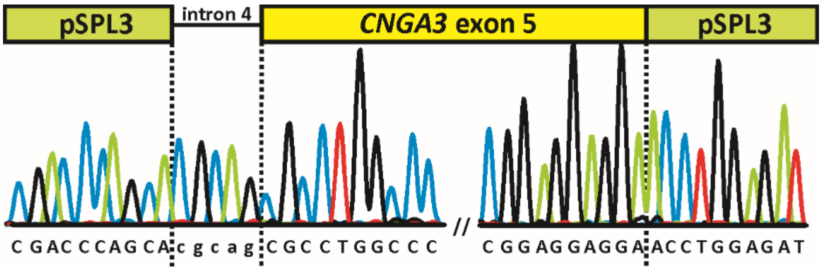

Figure 3A transcript 1: Correct splicing (exon 6 wild-type and c.450-15T>G minigenes)

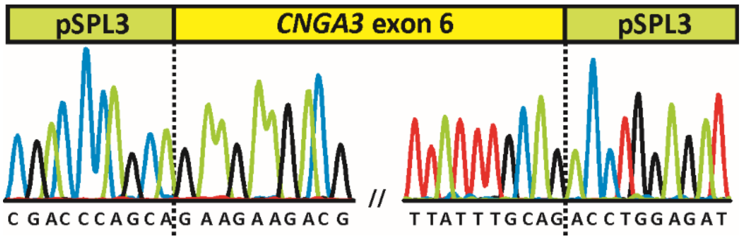

Figure 3A transcript 1: Correct splicing (c.566G>A minigene)

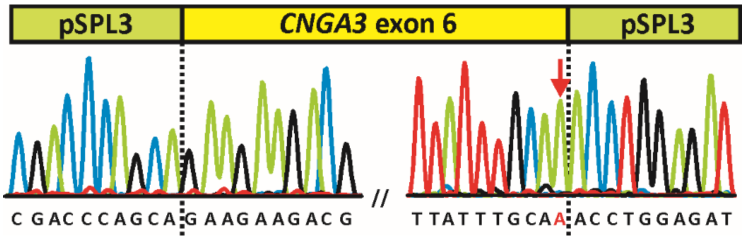

Figure 3A transcript 2: Retention of the last 14 nucleotides of intron 5 (c.450-15T>G minigene)

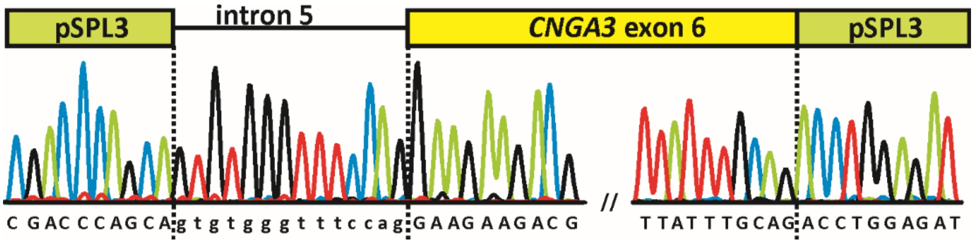

Figure 3A transcript 3: Retention of the last 49 nucleotides of intron 5 (c.450-15T>G minigene)

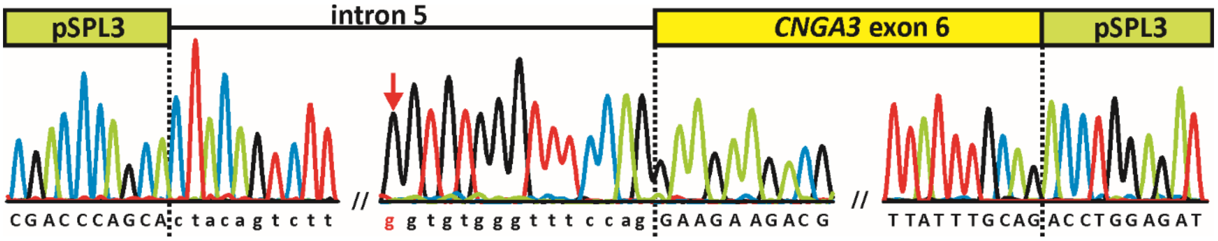

Figure 3A transcript 3: Retention of the last 49 nucleotides of intron 5 (c.450-1G>A minigene)

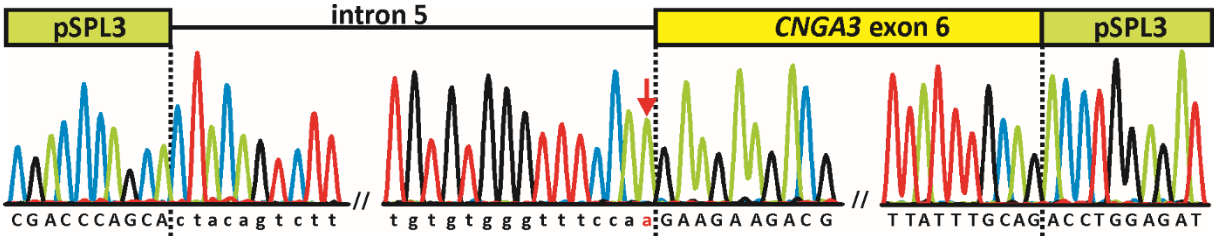

Figure 3A transcript 4: Skipping of exon 6 (c.450-15T>G and c.450-1G>A minigenes)

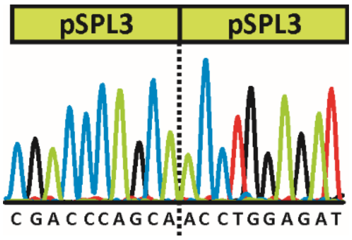

Figure 3A transcript 5: Retention of the last 154 nucleotides of intron 5 (c.450-1G>A minigene)

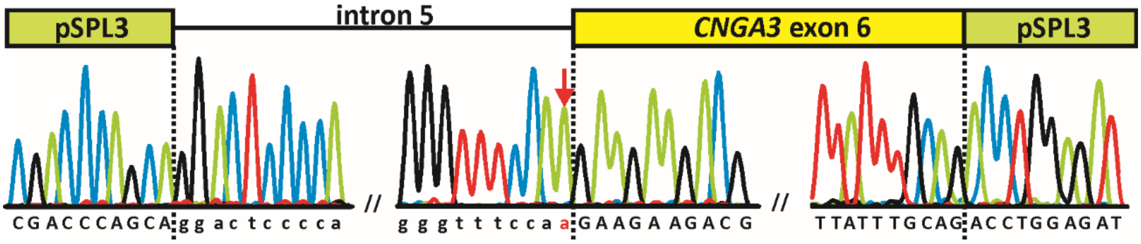

Figure 3A transcript 6: Retention of the last 70 nucleotides of intron 5 (c.450-1G>A minigene)

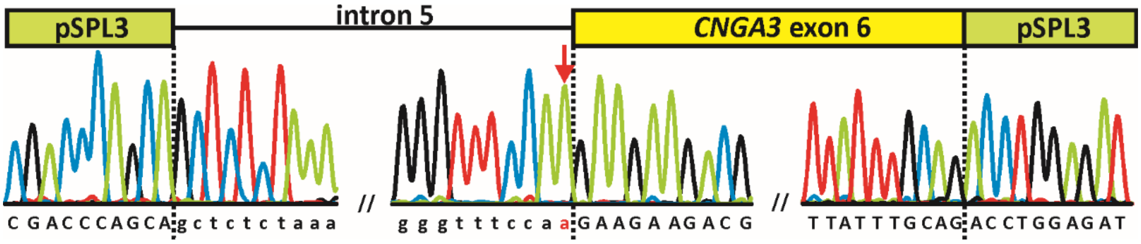

Figure 3A transcript 7: Deletion of the last 18 nucleotides of exon 6 (c.566G>A minigene)

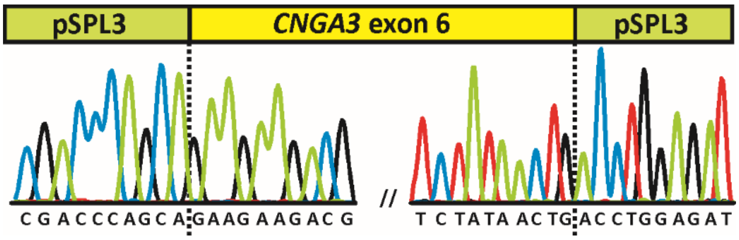

Figure 3B transcript 1: Correct splicing (exon 7 wild-type and c.673+5G>T minigenes)

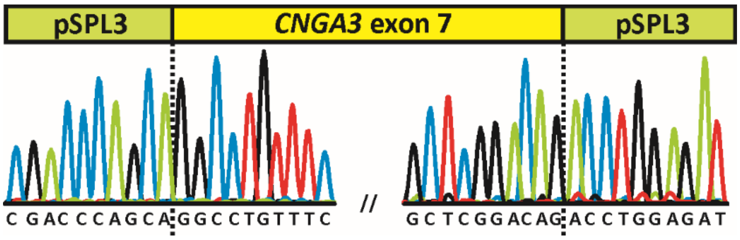

Figure 3B transcript 2: Retention of the last 9 nucleotides of intron 6 (c.567-11G>A minigene)

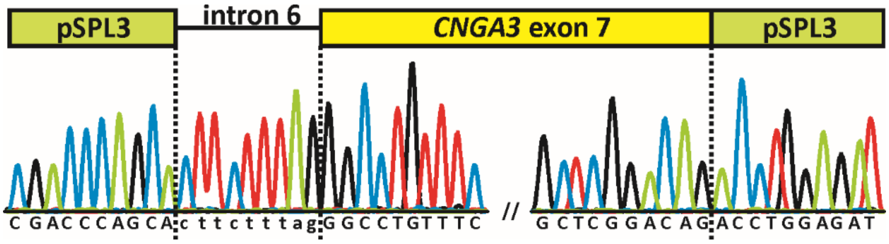

Figure 3B transcript 3: Skipping of exon 7 (c.673+5G>T minigene)

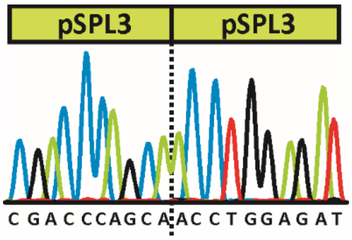

Figure 3C transcript 1: Correct splicing (exon 8 wild-type minigene)

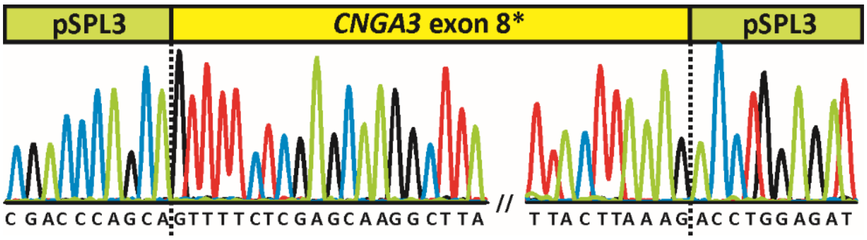

Figure 3C transcript 2: Deletion of the first 15 nucleotides of exon 8 (c.674-2A>C minigene)

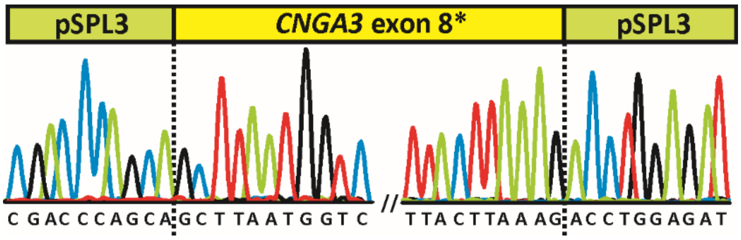

Figure 3C transcript 3: Deletion of the first 9 nucleotides of exon 8 (c.674-2A>C minigene)

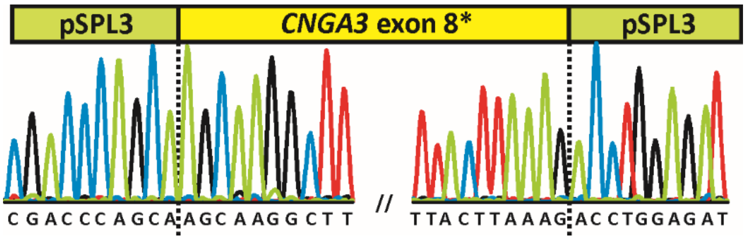

*Note that the minigene construct for exon 8 does not comprise the full-length exon.

Figure legends

Supplementary Figure S1: Minigene assays of variants that showed no splice defect.

The agarose gel (uncropped image) shows the RT-PCR products obtained upon transfection of HEK293T cells with the wildtype (WT) minigene constructs for *CNGA3* exon 3 (lane 2), exon 4 (lane 5), exon 5 (lane 7), exon 6 (lane 10), and exon 7 (lane 13). Also shown are the RT-PCR products from the mutant minigene constructs harboring variants c.211G>A (lane 3), c.215+11A>G (lane 4), c.395+9C>T (lane 6), c.396-4G>A (lane 8), c.449+13A>G (lane 9), c.566+6C>T (lane 11), c.566+14G>A (lane 12), c.670A>G (lane 14), c.671C>G (lane 15), and c.671C>T (lane 16). A size standard (low molecular weight DNA ladder, NEB) is loaded in the leftmost lane. RT-PCRs from transfection with empty pSPL3 vector (lane 17) and untransfected HEK293T cells (lane 18) served as controls. NRT (lane 19), no reverse transcriptase control; NTC (lane 20), no template control. Sequence electropherograms below the agarose gel show correct splicing (i.e., splicing of the respective *CNGA3* exon between the pSPL3 resident exons) for all minigene constructs. Note that only one representative sequence electropherogram is shown for minigenes harboring the same exon.

Supplementary Figure S2: Splice products of *CNGA3* variants inducing missplicing. Sequence electropherograms showing the splice junctions of all subcloned transcripts presented in Figure 2 and Figure 3. The pSPL3 exons are depicted in green, the *CNGA3* exons in yellow and intronic regions as a

black line. Variants are indicated by a red arrow if present in the corresponding transcript. Exonic nucleotides are shown in upper case and intronic nucleotides in lower case.
